# PIM1 signaling in immunoinflammatory diseases: an emerging therapeutic target

**DOI:** 10.3389/fimmu.2024.1443784

**Published:** 2024-09-20

**Authors:** Xue Yang, Chunming Liu, Yuxi Lei, Zhi Liu, Bin Zhu, Dongchi Zhao

**Affiliations:** ^1^ Department of Pediatrics, Xiangyang Central Hospital, Affiliated Hospital of Hubei University of Arts and Science, Xiangyang, Hubei, China; ^2^ Department of Pediatrics, Children’s Digital Health and Data Center, Zhongnan Hospital of Wuhan University, Wuhan, Hubei, China

**Keywords:** PIM1, serine/threonine protein kinase, immunoinflammatory disease, inflammatory bowel disease, rheumatoid arthritis

## Abstract

PIM1, the proviral integration site for Moloney murine leukemia virus, is a member of the serine/threonine protein kinase family. It is involved in many biological events, such as cell survival, cell cycle progression, cell proliferation, and cell migration, and has been widely studied in malignant diseases. However, recent studies have shown that PIM1 plays a prominent role in immunoinflammatory diseases, including autoimmune uveitis, inflammatory bowel disease, asthma, and rheumatoid arthritis. PIM1 can function in inflammatory signal transduction by phosphorylating multiple inflammatory protein substrates and mediating macrophage activation and T lymphocyte cell specification, thus participating in the development of multiple immunoinflammatory diseases. Moreover, the inhibition of PIM1 has been demonstrated to ameliorate certain immunoinflammatory disorders. Based on these studies, we suggest PIM1 as a potential therapeutic target for immunoinflammatory diseases and a valid candidate for future research. Herein, for the first time, we provide a detailed review that focuses on the roles of PIM1 in the pathogenesis of immunoinflammatory diseases.

## 1 Introduction

The proviral integration site for Moloney murine leukemia virus (PIM) is a family of serine/threonine protein kinases that include PIM1, PIM2, and PIM3. They are abnormally expressed in many oncologic diseases and have become an important therapeutic target for cancer (1). Among them, PIM1 was the first member to be discovered and has been studied the most. It is involved in various biological processes, such as cell survival, the cell cycle, cell proliferation and migration (2–4). Although PIM1 has been established to have a role in cancer progression, there is increasing evidence for wider pathological roles of PIM1 within the context of immunoinflammatory diseases, including Lupus nephritis (LN) and rheumatoid arthritis (RA) (5–7).

**Figure 1:**
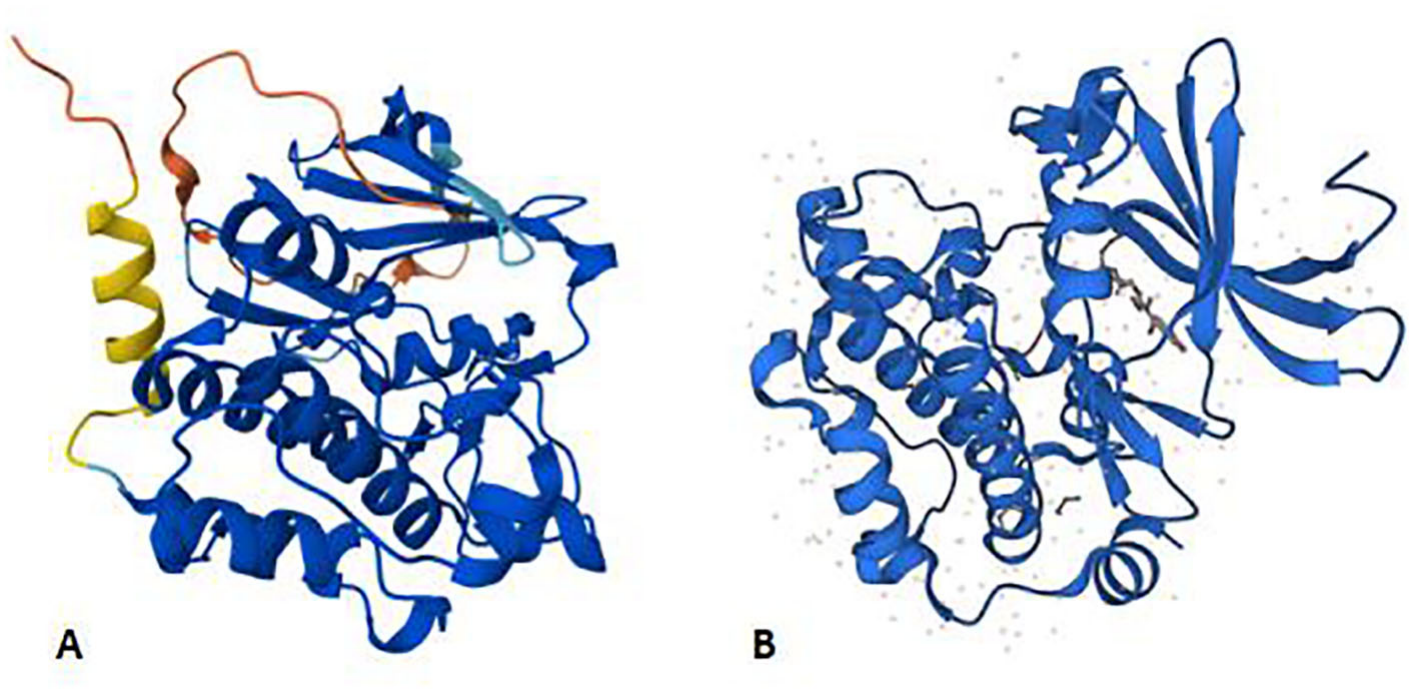
Panels **(A, B)** shows the AlphaFold structure prediction of murine PIM1 and human PIM1, respectively. Both of them consist of 313 amino acids. AlphaFold produces a per-residue model confidence score (pLDDT) between 0 and 100 to represent the model confidence. Very high (pLDDT > 90) High (90 > pLDDT > 70) Low (70 > pLDDT > 50) Very low (pLDDT < 50).

The PIM gene is a proto-oncogene encoding a serine/threonine protein kinase. The oncogene PIM1, which has an open reading frame and encodes a protein of 313 amino acids, is frequently activated by provirus insertion in murine leukemia virus-induced T-cell lymphomas. The structure prediction of PIM1 is shown in **Figure 1**. It spans six exons and is preceded and followed in all reading frames by stop codons (8). The murine PIM1 gene encodes a 44 kDa protein (PIM1L) and a 34 kDa protein (PIM1S) (9). PIM1L is synthesized via alternate translation starting at an upstream CUG codon and is the amino-terminal extension of PIM1S (10). The nucleotide AUG at positions 431–433 is the start codon for PIM1S, and the nucleotide CUG at sites 158–160 is the start codon for PIM1L (11). The expression of PIM1, irrespective of patient age or sex, is widespread and ranges from the haematopoietic and lymphoid system to the synovium and kidney (7, 9, 12).

Protein kinases can catalyse phosphorylation, which is one of the most significant posttranslational modifications of proteins and has been demonstrated to be a basic regulatory mechanism of cellular processes, including signal transduction, autophagy, and cell fate determination (4). Targeting the specific kinases that regulate the production of inflammatory mediators is one of the main treatment methods used to alleviate dysregulated inflammation in immunoinflammatory diseases (13). As a member of the serine/threonine protein kinase family, PIM1 plays a key role in inflammatory signal transduction through the phosphorylation of various inflammatory protein substrates, such as protein kinase B (AKT) and nuclear factor kappa-light-chain-enhancer of activated B cells (NF-κB), thereby mediating macrophage activation and T lymphocyte cell differentiation, which contributes to the occurrence and development of immunoinflammatory diseases (12, 14, 15). As expected, recent studies have shown the treatment effect of PIM1 inhibitors on multiple immunoinflammatory diseases. This review mainly analyses the key roles of PIM1 in the pathogenesis of immunoinflammatory diseases and reveals PIM1 as an emerging target for treating inflammatory disorders of the immune system.

## 2 Regulatory mechanism of PIM1

### 2.1 JAK/STAT signaling pathway

A variety of ligands, such as cytokines and growth factors, and their receptors activate the Janus kinase (JAK)/signal transducers of activated transcription (STAT) pathway (16). The four JAK proteins and the seven STAT proteins mediate intracellular signal transduction downstream of cytokine receptors, which participate in the pathology of allergic, autoimmune, and inflammatory diseases (17). Furthermore, abundant evidence has emerged in recent years that the overproduction of cytokines, especially interleukin-6 (IL-6), may cause the pathogenesis of some immunoinflammatory disorders (18). Therefore, the JAK/STAT signaling pathway and IL-6 have become attractive therapeutic targets for numerous immunoinflammatory diseases (19, 20).

**Figure 2:**
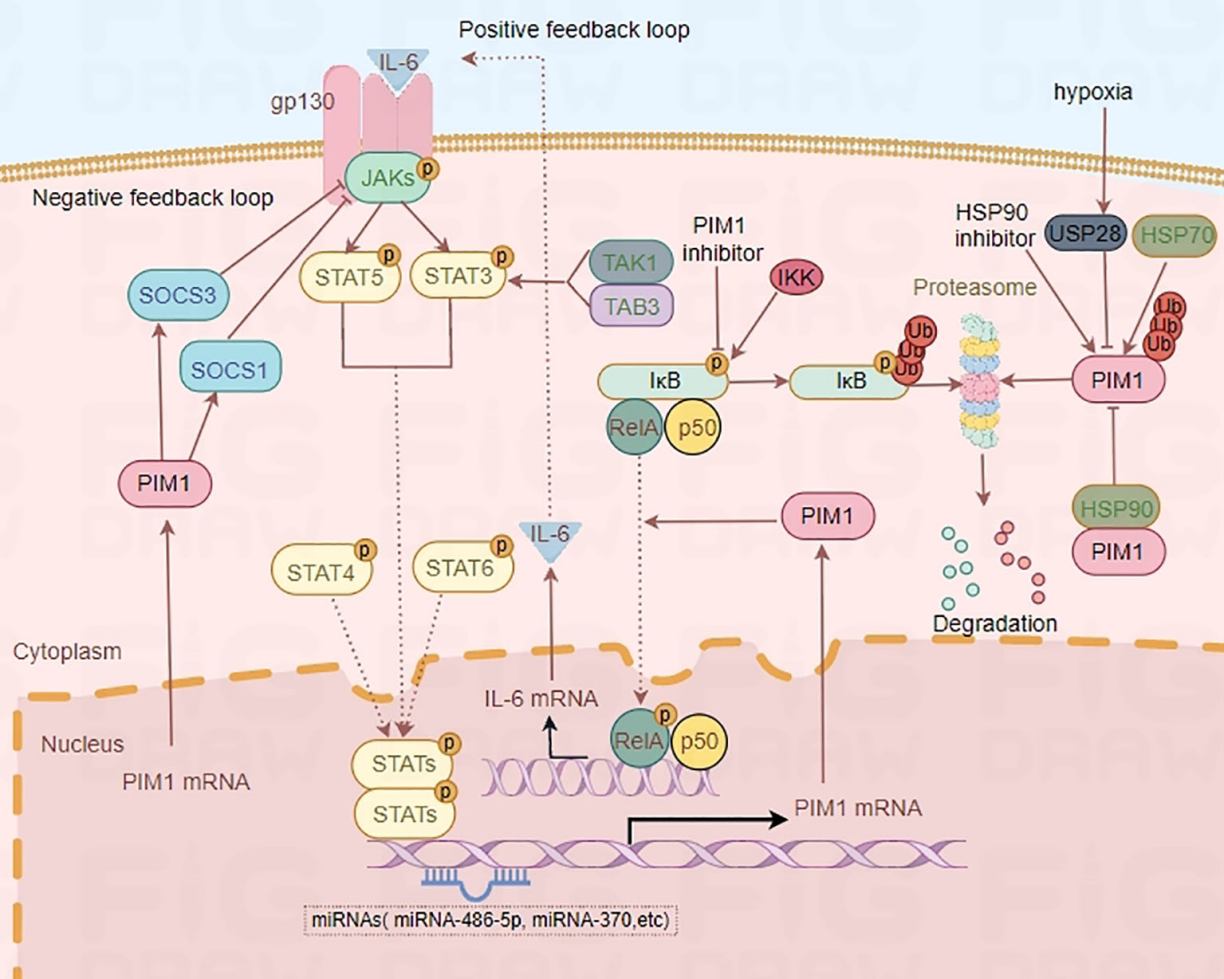
Regulatory mechanism of PIM1. IL-6-STAT3/5-PIM1 axis increases NF-κB activity and IL-6 production, which in turn enhances PIM1 expression and creates a positive feedback loop. However, PIM1 kinase can also modulate the JAKs/STAT3/5 signaling pathway by interacting with SOCS1 and SOCS3, and result in a negative feedback loop in JAKs-STAT3/5-PIM1 axis. Moreover, HSP90 and hypoxia-induced overexpression of USP28 can protect PIM1 from proteasomal degradation while HSP70 is associated with the degradation of PIM1 by ubiquitin.

Substantial evidence has shown that the JAK2/STAT3 pathway is one of the upstream regulatory pathways of PIM1 (21–23). Further study revealed that IL-6, a proinflammatory cytokine, can bind to its receptor and activate the JAK2/STAT3 signaling pathway, thus upregulating the expression of PIM1 (24, 25). Notably, PIM1 inhibitors can suppress the phosphorylation of IκB kinase, which contributes to the decreased NF-κB activity (26). Moreover, PIM1 has been demonstrated to phosphorylate p65/RelA, thereby increasing NF-κB activity and IL-6 production, which in turn enhances PIM1 expression and creates a positive feedback loop (27). In addition, transforming growth factor-β-activated kinase 1 (TAK1)-binding protein 3 (TAB3) regulates PIM1 expression by promoting STAT3 phosphorylation and activation through the formation of the TAB3–TAK1–STAT3 complex (28). In addition, PIM1 has already been revealed as one of the first known target genes of STAT5 (29, 30), and limited evidence indicates that STAT4 and STAT6 also regulate PIM1 expression (31). In contrast to the PIM1–NF-κB–IL-6 axis, PIM1 can modulate the JAK2/STAT3/5 signaling pathway by interacting with suppressor of cytokine signaling 1 (SOCS1) and SOCS3, resulting in a negative feedback loop in the JAK2-STAT3/5-PIM1 axis (**Figure 2**) (22, 32, 33). Overall, PIM1 plays an essential role in the crosstalk between the JAK2-STAT3/5 pathway and the NF-κB–IL-6 pathway, which are attractive targets for treating immunoinflammatory diseases.

### 2.2 MicroRNAs

MicroRNAs (miRNAs), which are single-strand endogenous and noncoding RNA molecules with a length of approximately 22 nucleotides, have emerged as a principal group of gene expression regulators related to a wide spectrum of biological processes through the modulation of the translation and stability of their target mRNAs (34). MiRNAs, which are crucial for the progression of immunoinflammatory diseases (35, 36), play a vital role in B- and T-lymphocyte development, megakaryocytopoiesis, monocytopoiesis, and granulopoiesis, as demonstrated in mice with deficient or overexpressed copies of particular miRNAs (37). In a preclinical study, miRNA-486-5p expression was found to be significantly correlated with the expression of IL-6, IL-8, tumor necrosis factor-α (TNF-α), and interferon-γ (IFN-γ). Further cell experiments revealed that miRNA-486-5p could target histone acetyltransferase 1 (HAT1) to regulate the Toll-like receptor 4 (TLR4)-triggered inflammatory response of alveolar macrophages (38). In addition, exosomal miRNA-486-5p derived from rheumatoid arthritis fibroblast-like synoviocytes (RA-FLSs) induces osteoblast differentiation through the Tob1/BMP/Smad pathway, contributing to the pathogenesis of RA (35). Accordingly, it can be inferred that upregulated miRNA-486-5p is involved in the inflammatory response and immunoinflammatory diseases. Many lines of evidence suggest that the proinflammatory factor PIM1 is a target gene of miRNA-486-5p, which can negatively regulate the expression of PIM1 through binding to the PIM1 mRNA 3’-UTR and mediate cellular oxidative stress, cell proliferation, and epithelial–mesenchymal transition (39–41). Therefore, we deduced that the miRNA-486-5p/PIM1 axis may play a role in the immunoinflammatory response. miRNA-370 was also found to act as a tumor suppressor that targets PIM1 gene expression, thus regulating glycolysis in hepatocellular carcinoma cell lines (42, 43). In addition to miRNA-486-5p and miRNA-370, other miRNAs, such as miRNA-33a, miRNA-328-3p, and miRNA-1294, have also been revealed to target the PIM1 gene directly (42–46). However, most of the miRNA/PIM1 axes have been studied in malignant conditions, and further studies are needed to determine the role of miRNAs/PIM1 in immunoinflammatory diseases.

### 2.3 HSP90 and the ubiquitin−proteasome pathway

Heat shock proteins (HSPs) are molecular chaperones that participate in the refolding of misfolded proteins, thus contributing to the maintenance of cellular homeostasis (47). These proteins can be classified into several families, such as HSP70, HSP90, HSP110, and chaperonin. HSP90 is involved in many cellular processes, such as apoptosis, autophagy, and the immune response, since it has abundant protein substrates (48, 49). The ubiquitin−proteasome pathway plays key roles in almost all cellular processes, in which proteins tagged with certain types of polyubiquitin chains are specifically recognized and removed by the proteasome (50). A study revealed that overexpressed deubiquitinase ubiquitin-specific protease 28 (USP28) in hypoxia condition regulated the stability of PIM1 by reducing its ubiquitination and proteasomal degradation (51). In addition, it has been reported that HSP90 and the ubiquitin−proteasome pathway jointly participate in regulating the stability of PIM1 (52). In that study, HSP90 was found to protect PIM1 from proteasomal degradation, while HSP70 was associated with the degradation of PIM1 by ubiquitin (52). Specifically, PIM1 physically interacts with HSP90α and HSP90β and is rapidly degraded upon exposure to the HSP90-specific inhibitor geldanamycin (53). Moreover, similar to PIM1, HSP90α expression is controlled by a signal from the cytokine receptor gp130. These findings suggest that HSP90 is coregulated with PIM1 and contributes to PIM1 stabilization and functionality (53).

## 3 The role of PIM1 in classical macrophage activation

Macrophages are heterogeneous cells that play a vital role in inflammatory and tissue reparative responses. Their activation or deactivation by different signals in the contexts of microorganisms, the tissue microenvironment, and cytokine signals partially imprints this diversity (54). There are two main polarization states of macrophages: classically activated macrophages (M1) and alternatively activated macrophages (M2), which display pro- and anti-inflammatory phenotypes, respectively. The polarization state of macrophages is dependent on molecules in the microenvironment, including several cytokines and chemokines (55). Numerous studies have shown that M1 macrophages can be activated by lipopolysaccharides (LPS) or T helper 1 (Th1) cell cytokines such as IFN-γ (56, 57). M1 macrophages can also produce proinflammatory cytokines such as IL-1β, IL-6, IL-23 and TNF-α, which are involved in Th1 and Th17 cell differentiation (58, 59), while disruption of this intricate crosstalk by blocking IL-6 or IL-23 function alters these conditions (60). In addition, classically activated macrophage-derived exosomes are also reported to aggravate immune-mediated disorders by boosting Th1 and Th17 responses (61). It follows that classical macrophage activation plays a key role in Th1 and Th17 differentiation, promoting chronic inflammation and the occurrence of immunoinflammatory disease.

PIM1 expression was found to be significantly induced in RAW264.7 cells activated by LPS (62), which may be attributed to the overproduction of IL-6 by activated macrophages. Many lines of evidence suggest that activation of the NF-κB pathway and the Nod-like receptor protein 3 (NLRP3) inflammasome promotes macrophage activation, while inhibition of these pathways can downregulate the M1 phenotype of macrophages and ameliorate inflammatory diseases (58, 63). PIM1, a downstream effector of many cytokine signaling pathways, plays a proinflammatory role in immunoinflammatory disease. It has been shown to activate NF-κB signaling by phosphorylating RelA/p65 Ser276 to defend against ubiquitin-mediated degradation and recruit RelA/p65 to kappa B-elements after TNF-α stimulation, while PIM1 knockdown weakens IL-6 production by interfering with RelA/p65 activation (64). Many studies have shown that PIM1 inhibitors such as SMI-4a and KMU-470 can also suppress macrophage inflammatory responses by inhibiting the upregulation of a major component of the inflammasome complex, NLRP3, and the phosphorylation of IκB kinase and NF-κB (26, 65). A recent study indicated that suppression of PIM1 by SMI-4a specifically blocks the oligomerization of apoptosis-associated speck-like protein containing a CARD (ASC) in the assembly stage and rapidly inhibits NLRP3 inflammasome activation in mouse and human macrophages (66). Taken together, these findings indicate that PIM1 plays a vital role in activating inflammatory signaling, which contributes to classical macrophage activation, and PIM1 inhibitors are potential anti-inflammatory agents for treating immunoinflammatory diseases.

## 4 The role of PIM1 in CD4+ T-cell differentiation

T-cell differentiation is a highly regulated, multistep process that is essential for the immunological response. Once activated, naïve CD4+ T cells differentiate into distinct cytokine-producing Th subsets. By producing lineage-specific cytokines, effector Th subsets play significant roles in regulating immune responses and are involved in the pathogenesis of many immunoinflammatory diseases, including autoimmunity, allergies, and asthma (67).

**Figure 3:**
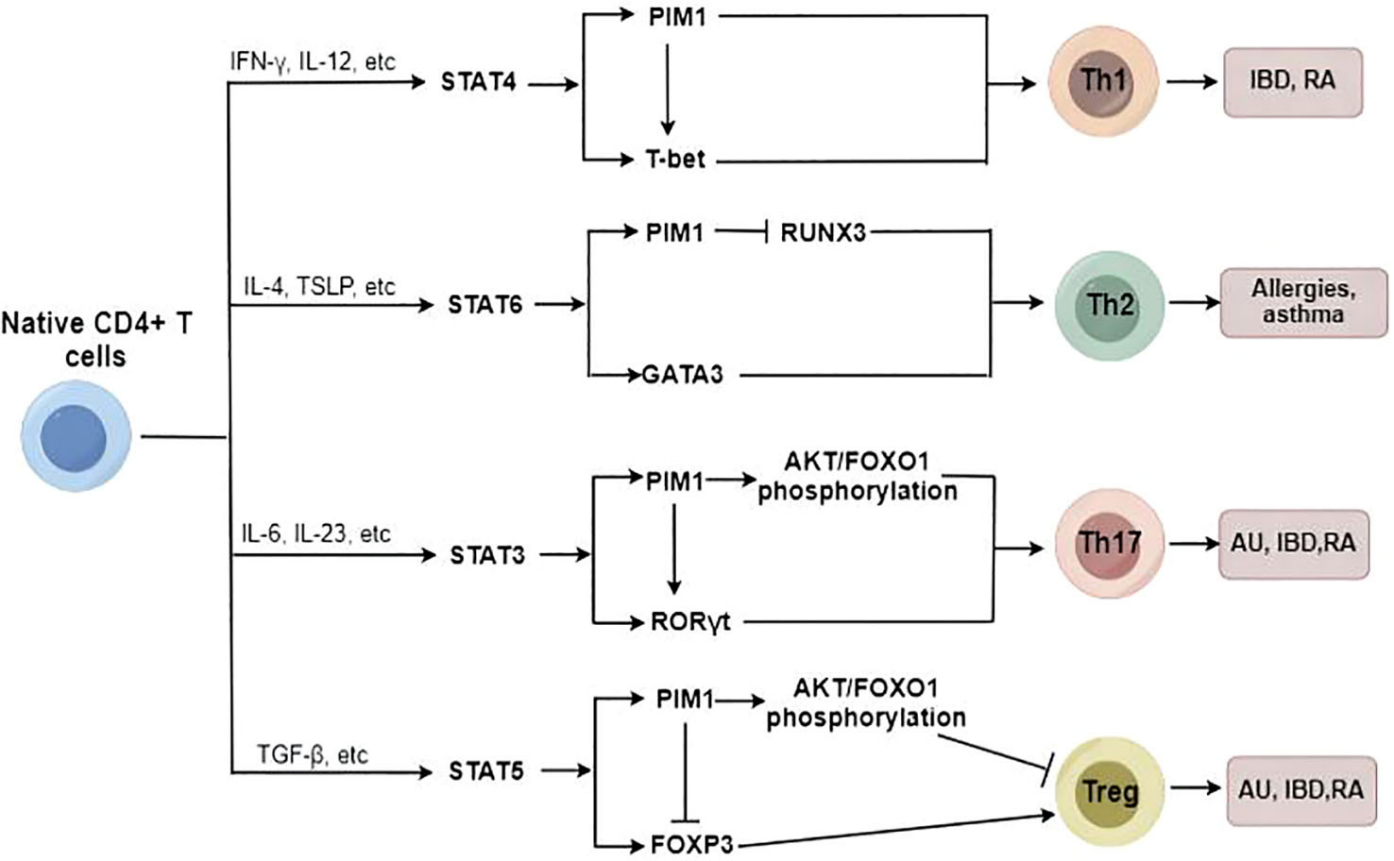
The potential role of PIM1 in Native CD4+ T cell differentiation. PIM1 plays an essential role in promoting Th17/Th1/Th2 cell differentiation and inhibiting Treg cell differentiation under abnormal immunoinflammatory conditions.

Accumulating evidence has shown that inhibiting PIM1 reduces the proportion of Th17 cells and increases the proportion of regulatory T cells (Tregs) in patients with immunoinflammatory diseases (15, 62), likely due to the regulatory effects of PIM1 on the AKT/Forkhead box O1 (FOXO1) signaling pathway and Forkhead box protein 3 (FOXP3) phosphorylation (**Figure 3**) (14, 15). Moreover, as we mentioned before, PIM1 plays a vital role in classical macrophage activation, which also contributes to Th1 and Th17 differentiation. However, Buchacher et al. demonstrated that PIM kinases inhibit early Th17 cell differentiation and control the Th1/Th17 axis in healthy individuals, potentially via STAT1 and STAT3 (68). This contradiction may be attributed to the different stages of Th differentiation and the different health conditions of individuals.

In addition, PIM1 plays a critical role in regulating Th2 cell differentiation, and inhibition of PIM1 suppresses Th2 cell differentiation by enhancing Runt-related transcription factor 3 (RUNX3) expression (69). In addition to regulating Th2/Th17/Treg cell differentiation, PIM1 also acts as a potent promoter of Th1 cell differentiation. The PIM1 protein levels can be strongly upregulated by Th1-specific cytokines such as IL-12 and IFN-α in immunocytes (70), and PIM1 accordingly is involved in regulating IL-12/STAT4 signaling, which is a critical pathway in Th1 cell differentiation (31). Therefore, PIM1 inhibition has been identified as a protective measure to reduce the proinflammatory immune response through the downregulation of excessive Th1-type immune responses (62).

Overall, PIM1 plays an essential role in promoting Th17/Th1/Th2 cell differentiation to varying degrees and inhibiting Treg cell differentiation under abnormal immunoinflammatory conditions; thus, targeting PIM1 has the potential to attenuate the imbalance of T-cell subsets and ameliorate immunoinflammatory diseases.

## 5 The relationship between PIM1 and immunoinflammatory diseases

### 5.1 PIM1 and AU

Autoimmune uveitis (AU), a sight-threatening disease affecting ocular tissues such as the ciliary body, vitreous body, choroid and retina, is initiated by the aberrant activation of CD4+ T cells and subsequent recruitment of inflammatory cells (granulocytes, monocytes/macrophages) to the eyes, which ultimately causes tissue impairment and potentially leads to blindness (71, 72). Strikingly, enhanced pathogenicity of Th17 cells and an imbalance of the Th17/Treg axis have been identified as key reasons for the pathogenesis of AU (73–75). In addition, an imbalanced Th1/Th2 ratio, caused by activation of the Notch signaling pathway, also contributes to the occurrence of AU (75). Thus, modulating CD4+ T-cell homeostasis may serve as a measure to prevent abnormal immunoinflammatory responses and improve the pathological condition of AU. Accumulating evidence suggests that the phosphoinositide 3 kinase (PI3K)/AKT/FOXO1 signaling pathway plays an important role in regulating Th17/Treg homeostasis in autoimmune diseases (76–78). Specifically, AKT can regulate T-cell differentiation through the negative regulation of FOXO1. When FOXO1 is located in the nucleus, it enhances the function of Treg cells by maintaining the expression of FOXP3 but inhibits the differentiation of Th17 cells by inhibiting the expression of retinoid-related orphan receptor gamma t (RORγt) (79–81). However, phosphorylated AKT can directly phosphorylate FOXO1, thereby inducing FOXO1 to leave the nucleus. It becomes sequestrated in the cytosol and loses its regulatory function in Th17 cells and Treg cells (81, 82). Therefore, it is plausible that restoring proper regulation of the AKT/FOXO1 pathway may serve as a potential measure to facilitate Th17/Treg homeostasis in AU.

Recent studies have revealed the upregulation of PIM1 in T-cell subsets in animal models of experimental AU (15, 83). In addition, human uveitis, Vogt−Koyanagi−Harada disease (VKH), is characterized by the overexpression of PIM1 in CD4+ T cells and plasma cells, and PIM1 inhibition decreases the expansion of CD4+ T and B cells (15). Rac1 and the inhibitor of differentiation (ID2)/PIM1 axis were found to potentiate the pathogenicity of Th17 cells during EAU (74). Kurarinone, a major component of the traditional Chinese medicine Sophorae Flavescentis Radix, can regulate the Th17/Treg balance and ameliorate AU via Rac1 and ID2/PIM1 axis inhibition (74). In another recent study, progesterone was also found to improve the Th17/Treg imbalance by reversing the ID2/PIM1 axis, thereby alleviating the pathogenicity of Th17 cells in AU (84).

**Figure 4:**
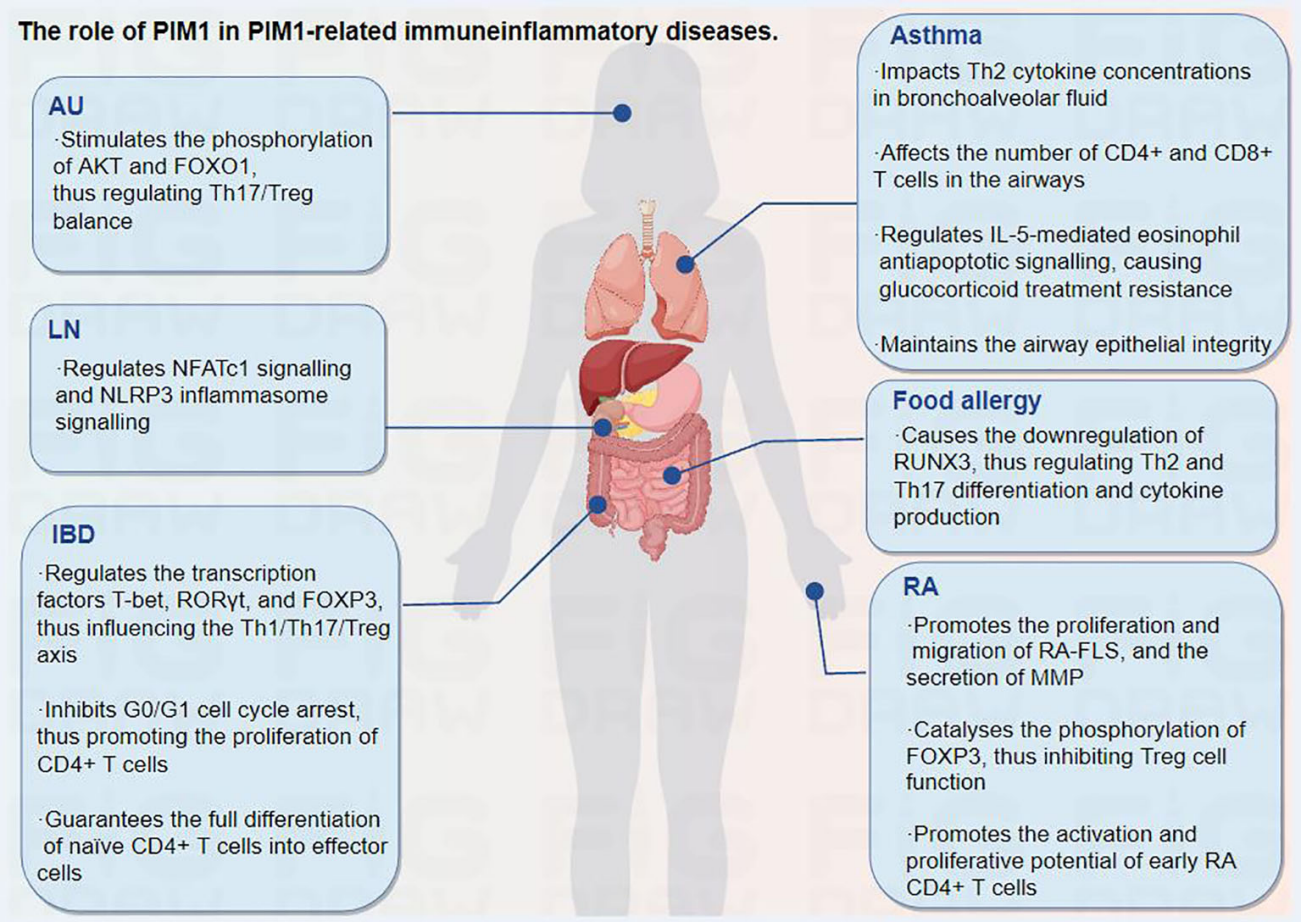
The role of PIM1 in PIM1-related immune inflammatory diseases.

Moreover, the immunosuppressor mycophenolate mofetil can improve abnormal immune cell activation in AU by reducing the expression of several pathogenic factors, including PIM1 (83). In addition, a recent multicellular immunokinetic study reported that PIM1 can promote an imbalance of Th17 and Treg cells, while inhibition of PIM1 activity reduces the ratio of Th17 cells and increases the ratio of Treg cells, thereby restoring the Th17/Treg cell balance in AU (15). Further study revealed that PIM1 can stimulate the phosphorylation of AKT and FOXO1 in total CD4+ T cells, resulting in an abnormal AKT/FOXO1 pathway and contributing to the Th17/Treg cell imbalance in AU, which can be ameliorated by PIM1 inhibition (**Figure 4**) (15). Overall, PIM1, which is aberrantly expressed in AU, is involved in mediating the Th17/Treg imbalance, and targeting PIM1 may be a promising therapeutic approach for AU.

### 5.2 PIM1 and IBD

Inflammatory bowel disease (IBD), including Crohn’s disease and ulcerative colitis, refers to a group of refractory and chronic inflammatory disorders of the gastrointestinal tract that are primarily diagnosed during adolescence and young adulthood, with an increasing incidence in pediatric populations (85). Therefore, IBD has been increasingly recognized as a serious, worldwide health-care problem. IBD is thought to be caused by an aberrant and continuing mucosal immune response to the intestinal microflora, strongly affected by a genetic predisposition of the individual (85, 86). Numerous studies have shown that IBD development is accompanied by an enhanced immune response and decreased immune tolerance, which results from immune activation of Th17 and Th1 cells and a reduction in the number of Treg cells (87–89). Dysregulation of this balance between effector T-cell subsets, especially the Th17/Treg axis, leads to chronic inflammation and autoimmunity in IBD (89).

It has been revealed that PIM1 expression is positively correlated with the degree of intestinal inflammation in experimental murine IBD models, suggesting that PIM1 signaling participates in the intestinal inflammatory response (90). Further studies revealed that inhibition of PIM1 reduces the mRNA and protein expression of transcription factors, including T-box expressed in T cells (T-bet) and RORγt, which are important for Th1 and Th17 differentiation from naïve T cells, respectively, and increases the mRNA and protein levels of the specific expression factor FOXP3 in Treg cells, thus improving the imbalance of T-cell subsets and attenuating mucosal inflammatory damage (62, 91, 92).

Moreover, classically activated macrophages play pivotal roles in IBD, and PIM1 inhibitors can reduce the proinflammatory response in IBD by inhibiting the hyperactivation of macrophages (62). In addition, the inhibition of PIM1 results in G0/G1 cell cycle arrest, which inhibits the proliferation of CD4+ T cells without compromising their ability to survive (93). Naïve CD4+ T cells are unable to fully differentiate into effector cells *in vitro* and *in vivo* in the absence of PIM1 kinase activity. Thus, PIM1 kinase activity may contribute to sustained disease severity, as evidenced by the successful use of a PIM1 inhibitor as a therapeutic measure in an IBD model mediated by CD4+ T cells (93). In general, PIM1 plays an important role in CD4+ T-cell proliferation and differentiation, and inhibition of PIM1 kinase activity may provide therapeutic benefit in IBD.

### 5.3 PIM1 and RA

RA is a chronic, autoimmune inflammatory disease that mainly impacts the joints and periarticular soft tissues, leading to joint inflammation, destruction, and even disability (94). To date, there is no cure for RA, and the treatment effect varies across patients, indicating an undetermined pathogenic diversity (95). Nonetheless, early and accurate diagnosis is still crucial for optimal therapeutic success in RA, especially for patients with high risk factors for a poor prognosis. The immune response composition of RA synovial tissue includes congenital (e.g., monocytes, dendritic cells, innate lymphocytes) and adaptive (e.g., Th1, Th17, B cells, and plasma cells) immune responses, as well as strong tissue inflammatory responses that aggravate joint destruction (95, 96).

Excessive STAT3 activation and dysregulated STAT3 signaling in CD4+ T cells have been proposed as early pathophysiologies in the clinical phase of RA (97). A clinical study demonstrated that the STAT3 target genes PIM1, B-cell leukemia-3 (BCL3), and SOCS3 in peripheral blood CD4+ T cells from treatment-naïve RA patients show the strongest differential regulation, with each gene’s expression highly associated with paired intracellular phospho-STAT3 (5). Furthermore, in a meta-analysis of 279 patients, the majority of the signature’s capacity to distinguish between RA patients was mediated via the same three genes, independent of age, joint involvement, or acute phase response (5). It follows that the dysregulation of PIM1, SOCS3 and BCL3 in circulating CD4+ T cells, which is mediated by aberrant STAT3 activation, is a distinguishing feature of early RA that occurs independently of the acute phase response. As such, PIM1 is also upregulated in the RA synovium, T cells, macrophages and RA-FLSs (12).

Moreover, significant suppression of RA-FLS proliferation, migration, and matrix metalloprotease (MMP) secretion from stimulated RA-FLSs was found upon PIM1 knockdown or AZD1208 administration. The phosphorylation levels of cyclic adenosine monophosphate (cAMP) response element binding protein and extracellular signal-regulated kinase in RA-FLSs were notably affected by PIM1 knockdown (12). These results suggest that PIM1 can serve as a novel regulator of the invasive and aggressive behavior of RA-FLSs, which indicates its potential as a target for RA treatment.

PIM1 has been implicated in the cytokine-dependent proliferation and survival of T lymphocytes (31). In addition, PIM1 can specifically catalyse the phosphorylation of the FOXP3 Ser422 site, thus negatively regulating the transcriptional activity of FOXP3 and inhibiting Treg cell function (14). A recent clinical study showed that PIM1 levels are upregulated in synovial CD4+ T cells from patients with early RA. Further ex vivo research indicated that the activation and proliferative potential of early RA CD4+ T cells stimulated by the overproduction of proinflammatory cytokines such as IFN-γ and IL-17 and the reduced proportion of FOXP3+ Treg cells are reversed by exposure to PIM1 kinase inhibitors, thus effectively alleviating arthritis progression and cartilage destruction (98). In addition, a dual IL-1 receptor-associated kinase-4 (IRAK4)/PIM1 inhibitor has also been found to function by blocking TLR/myeloid differentiation factor-88 (MYD88)-mediated crosstalk of NF-κB activation, inhibiting the JAK/STAT pathway and PIM1 expression, thus improving RA conditions (6). Taken together, PIM1 plays a critical role in regulating the invasive and aggressive behavior of RA-FLSs and diagnosing RA in the early phase, which indicates its great potential as a target for RA treatment.

### 5.4 PIM1 and allergic diseases

Allergic diseases, which include eczema, atopic dermatitis, allergic asthma, and food allergy, are systemic disorders resulting from an impaired immune system. The constantly rising incidence rates of these disorders, along with their high recurrence rates, are creating a significant medical and socioeconomic burden and drawing increasing amounts of attention. Many factors, including the living environment, the maternal-foetal environment, genetics, epigenetics, and the immune system, play complex roles in the pathogenesis of allergic illnesses (99).

Asthma, one of the most common chronic, noncommunicable diseases in children and adults, is characterized by variable restriction of airflow and leads to variable respiratory symptoms (100). The development of allergic asthma is influenced mostly by allergen-specific Th2 cells and pulmonary Th2 cell responses (101), and glucocorticoid administration is an effective treatment for steroid-sensitive asthma (102). It has been revealed that pulmonary PIM1 concentrations are increased in mouse models of asthma after ovalbumin sensitization and challenge (103). Moreover, inhibition of PIM1 kinase activity prevents the development of goblet cell metaplasia, eosinophilic airway inflammation, and airway hyperresponsiveness and increases Th2 cytokine concentrations in bronchoalveolar fluid in a dose-dependent manner, revealing the key role of PIM1 in the full development of allergen-induced airway responses. In addition, a significant reduction in the number of CD4+ and CD8+ T cells and in the concentrations of cytokines have also been found in the airways (103).

CD4+ and CD8+ effector T cells are involved in the development of allergic inflammation, and PIM1 has been found to enhance the activity of nuclear factor of activated T cells (NFATc) and the production of IL-2, promoting the proliferation and survival of IL-2-dependent T lymphocytes (103, 104). Moreover, inhibition of PIM1 kinase activity alleviates the immune response by limiting the activation and proliferation of CD4+ and CD8+ T cells (98, 103, 104). PIM1 also plays a major role in IL-5-mediated eosinophil antiapoptotic signaling, causing glucocorticoid treatment resistance, while blocking PIM1 signaling restores glucocorticoid receptor-mediated apoptosis in IL-5-activated eosinophils, thereby ameliorating glucocorticoid resistance in eosinophil asthma (105). However, it has also been reported that PIM1 kinase activity can help maintain airway epithelial integrity and prevent airway epithelial inflammatory activation to exert a beneficial effect in a house dust mite-driven model (HDM) of allergic asthma (106). Overall, the inhibition of PIM1 seems to be more beneficial than harmful in treating asthma. Targeting PIM1 may be effective in preventing the development of airway hyperresponsiveness, airway inflammation, and cytokine production in patients with asthma.

Food allergy refers to adverse food reactions mediated by immunological mechanisms, namely, abnormal or excessive immune reactions caused by food proteins, which can be mediated by IgE or non-IgE. Among them, peanuts are important food allergens that cause severe allergic reactions. Furthermore, unlike many other food allergies, peanut allergies typically persist into adulthood (107). Th2 cell differentiation and the release of IL-4 and IL-13 drive IgE-mediated activation of mast cells, which are involved in food allergic reactions (108). The activation of PIM1 plays an essential role in the development of peanut-induced intestinal anaphylaxis (69, 108). Specifically, PIM1 kinases can phosphorylate RUNX family members, enhance their activity, and regulate their expression (69, 109). Among the RUNX family members, RUNX3 is a known silencer of the IL-4 gene locus, which inhibits IL-4 production in T cells and regulates T-cell differentiation via a physical interaction with NFAT (110). The activation of PIM1 leads to the downregulation of RUNX3, while the inhibition of PIM1 can reduce Th2 and Th17 differentiation and cytokine production through the upregulation of RUNX3, thus attenuating intestinal allergic inflammation (69, 108). Collectively, this evidence suggests that targeting the PIM1/RUNX3 axis may be a novel potential measure for the control of food-induced allergic reactions through the regulation of Th2 and Th17 differentiation.

### 5.5 PIM1 and LN

LN is one of the most serious and common complications of systemic lupus erythematosus (SLE), which is a systemic autoimmune disease characterized by multiple organ involvement and a large number of complications. Long-term inflammation may cause irreversible damage to the kidney and may lead to chronic renal disease that can progress to end-stage renal disease. Activation of the NLRP3 inflammasome plays an important role in the pathogenesis of LN, while inhibition of the NLRP3 inflammasome provides a new strategy for LN treatment (111).

A clinical study revealed that PIM1 expression is upregulated in peripheral blood monocytes from patients with SLE and in renal biopsy tissue from patients with LN (7). Further *in vitro* and *in vivo* studies suggested that PIM1 can regulate the activation of the NLRP3 inflammasome by regulating intracellular Ca2+, while PIM1 inhibition suppresses NFATc1 signaling and NLRP3 inflammasome signaling in mouse and human podocytes, thus improving the clinical symptoms and pathological damage of LN (7). In addition, CCAAT/enhancer-binding protein beta (CEBPβ), a basic leucine zipper (bZIP) transcription factor involved in innate immunity, regulates NLRP3 inflammasome activation and participates in the pathogenesis of SLE (112). CEBPβ can bind to the PIM1 promoter and promote the transcriptional activity of PIM1, while CEBPβ knockdown downregulates PIM1 expression, inhibits NLRP3 inflammasome activation and cell pyroptosis, and reduces the secretion of the inflammatory factors IL-1β and IL-6. Correspondingly, the overexpression of PIM1 reversed all of these effects (112). Therefore, targeting the PIM1/NLRP3 pathway may have therapeutic potential for LN.

## 6 Discussion

PIM1 has been a research hotspot for neoplastic diseases in the past. However, recent studies have shown that PIM1 also plays a vital role in the occurrence and development of immunoinflammatory diseases. Immunoinflammatory diseases such as AU, IBD and RA are characterized by abnormal PIM1 expression, which can affect the development of inflammatory diseases by mediating inflammatory signaling pathways, classical macrophage activation, and imbalances between effector T cells. Research on PIM1 in immunoinflammatory diseases provides novel ideas for exploring the pathogenesis and therapeutic use of PIM1 in clinical immunoinflammatory diseases. Numerous studies have shown that targeting PIM1 may be an effective treatment for immunoinflammatory diseases. Targeting PIM kinases also presents a compelling approach since knock-down of PIM leads to non-fatal phenotypes *in vivo* (113) and pan-PIM inhibition appears to be generally tolerated in Phase I/II studies (114, 115). In addition, PIM1 has been shown to be a potential biomarker for evaluating the status of certain immunoinflammatory diseases, such as RA (5, 12). Despite much progress in discovering the role of PIM1 in the pathogenesis of immunoinflammatory diseases, the molecular mechanisms underlying its effects and signaling pathways are still poorly understood. The function of PIM1 in immunoinflammatory diseases must be fully elucidated. Moreover, most of the studies were conducted in pathological animal models. The expression of PIM1 is not clear in patients with some immunoinflammatory diseases, such as IBD and asthma. Therefore, there is a pressing need to conduct additional clinical studies to further validate the roles of PIM1 as well as the safety and effects of PIM1 kinase inhibitors in immunoinflammatory diseases.

## Glossary

AKT: Protein kinase B
ASC: Apoptosis-associated speck-like protein containing a CARD
AU: Autoimmune uveitis
BCL3: B cell leukemia-3
bZip: Basic leucine zipper
cAMP: Cyclic adenosine monophosphate
CEBPβ: CCAAT/enhancer-binding protein beta
FOXO1: Forkhead box O1
FOXP3: Forkhead box protein 3
HAT1: Histone acetyltransferase 1
HDM: House dust mite-driven model
HSPs: Heat shock proteins
IBD: Inflammatory bowel disease
ID2: Inhibitor of differentiation
IFN-γ: Interferon-γ
IL-6: Interleukin-6
IRAK4: IL-1 receptor associated kinase-4
JAK: Janus kinase
LN: Lupus nephritis
LPS: Lipopolysaccharides
M1: Classically activated macrophages
M2: Alternatively activated macrophages
MiRNAs: MicroRNAs
MMP: Matrix metalloprotease
MYD88: Myeloid differentiation factor-88
NFATc: Nuclear factor of activated T cells
NF-κB: Nuclear factor kappa-light-chain-enhancer of activated B cells
NLRP3: Nod-like receptor protein 3
PI3K: Phosphoinositide 3 kinase
PIM: Proviral integration site for Moloney murine leukemic virus
RA: Rheumatoid arthritis
RA-FLSs: RA fibroblast-like synoviocytes
RORγt: Retinoid-related orphan receptor gamma t
RUNX3: Runt-related transcription factor
SLE: Systemic lupus erythematosus
SOCS1: Suppressor of cytokine signaling 1
STAT: Signal transducers of activated transcription
TAK1: Transforming growth factor-β-activated kinase 1
TAB3: TAK1-binding protein 3
T-bet: T-box expressed in T cells
Th1: T helper 1 cell
TLR4: Toll-like receptor 4
TNF-α: Tumor necrosis factor-α
Tregs: Regulatory T cells
USP28: ubiquitin-specific protease 28
VKH: Vogt-Koyanagi-Harada disease
